# Chemotherapy‐Induced Cardiotoxicity With Frontline BV‐AVD for Hodgkin Lymphoma

**DOI:** 10.1155/crom/4690413

**Published:** 2025-12-16

**Authors:** Angelo Rizzolo, Caroline Michel, Nathalie A. Johnson

**Affiliations:** ^1^ Division of Hematology, Jewish General Hospital, Montreal, Quebec, Canada, jgh.ca; ^2^ Division of Cardiology, Jewish General Hospital, Montreal, Quebec, Canada, jgh.ca

## Abstract

Brentuximab vedotin (BV) with chemotherapy is a novel and an effective treatment for Hodgkin Lymphoma (HL) but is associated with increased rates of peripheral neuropathy and neutropenia. Since its increased use, novel adverse effects of the regimen have come to light, including unexpected toxicities. We report a case of BV‐chemotherapy–induced cardiotoxicity, highlighting the need for vigilant monitoring and further research into the risk factors, mechanisms, and long‐term safety profile of this therapy.

## 1. Introduction

Brentuximab vedotin (BV) in combination with chemotherapy is used to treat classical Hodgkin Lymphoma (cHL)[1–3]. It offers improved outcomes relative to its predecessors at the expense of higher rates of peripheral neuropathy (PN) and neutropenia. In fact, real‐world data suggest that PN may occur at a rate as high as 78%, and such adverse effects result in dose omission in up to 46% of patients [4]. Since its clinical adoption, new adverse effects have also surfaced including pneumotoxicity, colitis, and progressive multifocal leukoencephalopathy (PML). Postmarketing pharmacovigilance data suggest that cardiotoxicity may represent a significant adverse effect not detected in clinical trials [5, 6]. Here, we share a case of cardiotoxicity in a patient receiving BV‐chemotherapy for cHL.

## 2. Case Presentation

A 58‐year‐old, man with hypertension and a remote 5–10 pack‐year smoking history was diagnosed with Stage IVB cHL with lung and bone involvement. His international prognostic score (IPSS) was 3. Pretreatment transthoracic echocardiogram (TTE) was unremarkable with a left ventricular ejection fraction (LVEF) of 65%, global longitudinal strain (GLS) of −17.9%, and no evidence of pericardial or valvular disease. He was treated with BV (1.2 mg/kg), doxorubicin (25 mg/m^2^), vinblastine (6 mg/m^2^), and dacarbazine (375 mg/m^2^) (AVD) on Days 1 and 15 of each 28‐day cycle. On Days 1–3 after treatment days, the patient endorsed retrosternal chest pain at rest lasting ~5 min then spontaneously resolving. These episodes became more frequent with each cycle. At the end of Cycle 2, he was found to have asymptomatic sinus tachycardia of 130 beats per minute. Repeat TTE showed a LVEF of 50%. Interim‐PET scan after two cycles was consistent with complete response (CR). He proceeded to receive Cycles 3 and 4 of therapy with vinblastine omitted due to Grade 1 PN.

At the end of Cycle 4, the patient endorsed persistent tachycardia and chest pain. He was admitted to the hospital for further investigations. N‐terminal brain natriuretic peptide (NT‐proBNP) was 647 pg/mL and high‐sensitivity troponin T was 34 ng/L from 7 ng/L prior to Cycle 2 (local laboratory reference range = 0–15 ng/L). Biochemistry including electrolytes, serum creatinine, and liver transaminases were unremarkable. Hemoglobin was 10.2 g/dL, and total white blood cell count was 32.9×10^9^/L with neutrophilic predominance (absolute neutrophil count 20.8×10^9^/L). Platelets were within normal limits. Electrocardiogram revealed new inferior Q‐waves and another repeat TTE showed a LVEF of 45%–50% with a regional wall motion variability and GLS of −12.4%. Computed tomography of the chest with intravenous contrast was negative for pulmonary embolus. Cardiac magnetic resonance imaging (CMRI) revealed biventricular dysfunction with a LVEF of 35% with marked dysynchrony and regional variability, severe hypokinesis of the anterior and anteroseptal walls without evidence of infarction or myocarditis, and a right ventricular EF of 44%. Coronary angiography revealed two main abnormalities: (1) symptomatic coronary vasospasm; which improved after nitroglycerin administration and (2) mild nonobstructive coronary artery disease (CAD) of the mid left anterior descending (LAD) artery and mid right coronary artery (RCA). Altogether, the patient was diagnosed with a mild nonobstructive CAD, coronary vasospasm, and chemotherapy‐induced cardiomyopathy. He was treated empirically for this with a high‐potency statin, aspirin, valsartan, and amlodipine. A PET scan done after four cycles of therapy was consistent with CR. The patient received a cumulative doxorubicin dose of 100 mg/m^2^. He remains in CR at 9 months of follow‐up from initial diagnosis after receiving four cycles of treatment. His NT‐proBNP is now 202 pg/mL 6 months later. Surveillance cardiac imaging 6 months later revealed a LVEF of > 55% with no evidence of any new structural abnormality. Further cycles of BV‐chemotherapy were omitted due to these adverse effects.

## 3. Discussion

Here, we report a case of cardiotoxicity in a patient receiving frontline BV‐AVD for advanced‐stage cHL. He was found to have objective evidence of biventricular dysfunction, inferior Q‐waves, and coronary vasospasm. The case illustrates a significant toxicity that underscores the need for further investigation of its risk factors and mechanisms.

BV, vinblastine, and doxorubicin are all potentially contributory. Vinblastine inhibits microtubule formation and therefore inhibits cell division. It is associated with coronary syndromes as well as autonomic dysfunction. The latter can contribute to coronary vasospasm and the induction of malignant arrhythmias [7]. The molecular mechanisms for these adverse effects remain unclear. The cardiotoxic effects of doxorubicin are well‐established. The mechanism is inflammatory, driven by generation of reactive oxygen species and lipid peroxidation of the cell membrane of cardiomyocytes [8]. These can lead to direct mechanical or electrical toxicities that, when compounded, can lead to significant heart failure with reduced ejection fraction. The effect is dose‐dependent, cumulative and risk factors include advanced age, hypertension, diabetes, and cigarette smoking. Less than 1% of cases of anthracycline‐induced cardiomyopathy are acute (i.e. within the first doses of the drug) [8]. BV is a chimeric IgG1 anti CD30 antibody conjugated to monomethylauristatin A (MMAE), an inhibitor of the microtubule network [9]. The complex is internalized, processed by lysosomal enzymes and MMAE is delivered to the intracellular space. Antibody opsonization of tumor cells and release of free MMAE in plasma are also possible mechanisms of action that may promote inflammation within the tumor microenvironment [10]. The downstream signaling of CD30 involves activation of NF‐*κ*B; however, its role in cell‐signaling physiologically remains to be fully explored. Monoclonal antibodies such as vascular endothelial growth factor inhibitors (VEGFi) and rituximab (anti‐CD20) have been associated with coronary syndromes, thought to be driven by a direct endothelial damage leading to ischemia and thus contribute to ventricular dysfunction [11]. Given CD30′s role in cellular immunity, BV may induce cardiotoxicity via related pathways. MMAE, meanwhile, is a microtubule inhibitor and therefore shares the same anti‐neoplastic mechanism as vinblastine, which has known cardiotoxicity. Similar to vinca‐alkaloids, PN is one of BV′s most frequent and disabling side effects. It is unknown if BV‐neuropathy affects the autonomic nervous system and causes vasospasm, like vinblastine [12]. However, sinus tachycardia was observed more frequently in the BV arm of the AETHERA study and cardiac adverse‐events occurred among patients with known risk factors. Altogether, it follows that BV‐induced cardiac adverse effects may, in part be driven by similar mechanisms to other monoclonal antibodies and vinblastine owing to its mechanism of action. Dacarbazine has no known cardiotoxic effects.

Our patient received six total doses of BV, doxorubicin, and dacarbazine and two doses of vinblastine. Firstly, although vinca‐alkaloids are associated with coronary syndromes, our patient′s symptoms persisted during Cycles 3 and 4 of therapy, with vinblastine omitted. Secondly, anthracycline‐induced cardiotoxicity is made more likely in our patient by his smoking history and known hypertension but less likely by his age < 60 years, cumulative dose of 100 mg/m^2^ and the onset of cardiotoxicity within the first two cycles of therapy [8]. Lastly, BV is a novel antibody‐drug conjugate for which cardiac adverse effects are uncommon [12]. The present report is necessary to inform pharmacovigilance, as it has been done for other regimens previously. For example, ventricular arrhythmias were not reported as serious adverse effects in the trials that led to the approval of ibrutinib [13].

In conclusion, although BV‐AVD is an effective regimen for HL, it comes with added toxicities leading to dose‐modifications and interruptions. Therefore, our case highlights the need for prompt assessment of suspected toxicity, particularly among symptomatic patients and those with pre‐existing cardiovascular risk factors. The role of serial echocardiographic monitoring among patients on potentially cardiotoxic chemotherapy regimens may allow for earlier diagnosis and initiation of modern pharmacotherapy; however, this remains to be prospectively evaluated [14]. As alternative frontline agents become available, real‐world data are required to assess the safety of BV‐AVD such that clinicians can assist patients in making evidence‐based, personalized treatment decisions for this malignancy with curative potential.

## Disclosure

N.A.J has received consultant fees from Merck, Bristol Myers Squib, and Seattle Genetics.

## Conflicts of Interest

The authors declare no conflicts of interest.

## Funding

No funding was received for this manuscript.

## Data Availability

Data sharing is not applicable to this article as no datasets were generated or analyzed during the current study.
